# Complexity Analysis of Surface Electromyography for Assessing the Myoelectric Manifestation of Muscle Fatigue: A Review

**DOI:** 10.3390/e22050529

**Published:** 2020-05-07

**Authors:** Susanna Rampichini, Taian Martins Vieira, Paolo Castiglioni, Giampiero Merati

**Affiliations:** 1 Department of Biomedical Sciences for Health, Università degli Studi di Milano, 20133 Milan, Italy; susanna.rampichini@unimi.it (S.R.); giampiero.merati@unimi.it (G.M.); 2 Laboratorio di Ingegneria del Sistema Neuromuscolare (LISiN), Dipartimento di Elettronica e Telecomunicazioni, Politecnico di Torino, 10129 Turin, Italy; 3 PoliToBIOMed Lab, Politecnico di Torino, 10129 Turin, Italy; 4 IRCCS Fondazione Don Carlo Gnocchi, 20148 Milan, Italy; pcastiglioni@dongnocchi.it

**Keywords:** sEMG, approximate entropy, sample entropy, fuzzy entropy, fractal dimension, recurrence quantification analysis, detrended fluctuation analysis, correlation dimension, largest Lyapunov exponent

## Abstract

The surface electromyography (sEMG) records the electrical activity of muscle fibers during contraction: one of its uses is to assess changes taking place within muscles in the course of a fatiguing contraction to provide insights into our understanding of muscle fatigue in training protocols and rehabilitation medicine. Until recently, these myoelectric manifestations of muscle fatigue (MMF) have been assessed essentially by linear sEMG analyses. However, sEMG shows a complex behavior, due to many concurrent factors. Therefore, in the last years, complexity-based methods have been tentatively applied to the sEMG signal to better individuate the MMF onset during sustained contractions. In this review, after describing concisely the traditional linear methods employed to assess MMF we present the complexity methods used for sEMG analysis based on an extensive literature search. We show that some of these indices, like those derived from recurrence plots, from entropy or fractal analysis, can detect MMF efficiently. However, we also show that more work remains to be done to compare the complexity indices in terms of reliability and sensibility; to optimize the choice of embedding dimension, time delay and threshold distance in reconstructing the phase space; and to elucidate the relationship between complexity estimators and the physiologic phenomena underlying the onset of MMF in exercising muscles.

## 1. Introduction

### 1.1. General Aspects

The analysis of surface electromyography (sEMG) is widely used to characterize the electrical activity of muscle fibers during a contraction, both in isometric (force generation without changing the length of the muscles) and isotonic conditions (force generation by either lengthening [eccentric contraction] or shortening [concentric contraction] the muscles). Whatever the type of contraction, the prolongation of muscle contractions over time invariably causes the onset of muscle fatigue, defined as the inability to sustain force generation over time. To date, sEMG revealed that signs of muscle fatigue may manifest prior to the fatigue onset, suggesting the susceptibility of muscles to fatigue could be assessed noninvasively from the skin. These early signs of myoelectric alterations are often termed myoelectric manifestations of muscle fatigue (MMF) and are of utmost interest in physiology, pathophysiology, training and rehabilitation studies. However, from the first studies on sEMG analysis during fatiguing contractions it has become apparent that the sEMG signal shows a complex behavior, due to many concurrent factors. Therefore, in recent years, different complexity-based methods of analysis previously applied to physical and other biological time series have been tentatively applied to the sEMG, searching for new techniques to individuate early and efficiently the MMF onset during sustained isotonic and isometric muscle contraction.

In this review, we briefly describe what MMF is and how it has been assessed, we introduce sEMG as a tool to study the mechanisms underpinning muscle fatigue and explain the main linear and spectral methods to detect MMF in exercising muscles. Then, we review the principal complexity methods for sEMG analysis based on an extensive literature search over different databases to be maximally descriptive of all the methodology used, without further considerations on the methodological approach, experimental design, data analysis, and results. For each index of sEMG complexity, we provide a brief description of its meaning, the algorithm for its estimation, the typical parameters setting in sEMG analysis and the main articles employing it in investigating different muscles activations. The relationships reported in previous studies between each index and the physiological mechanisms underpinning muscular activation are propaedeutic to better understand the impact of MMF on each index. Indeed, muscular activation occurring at the beginning of a fatiguing contraction represents the preliminary phase of the fatigued condition. The main results obtained in studies on muscle fatigue are presented and finally, the interpretative theories hypothesized by the investigators are introduced without any personal endorsement but as an objective representation of the state of the art of this field of research. 

### 1.2. Muscle Fatigue

Muscle fatigue, a reversible reduction in force generation capacity, continues to generate great interest in the scientific community worldwide [1,2,3,4]. Its manifestation in several neuromuscular disorders [5] and its influence on sports performance [6] and rehabilitation [7] have led to deeply explore the underlying mechanisms of this phenomenon, which seem to be multifactorial. Beyond psychological aspects, many neuromuscular features ascribable to the central and peripheral nervous system head for electrochemical alterations. Following a classic two-domain concept, central and peripheral fatigue can be distinguished whenever the involved mechanism relates to the spinal and supra-spinal tract (central origin) or to structures distal to the neuromuscular junction (peripheral origin). 

At the central level, within the cerebral motor cortex fatigue causes the alteration of cells excitability, the inhibition of motor cortex output and the interruption of action potential conductions at axonal branching sites. As a consequence, the recruiting strategy of muscle fibers, based on increasing the number of muscle fibers and their discharge rate, is deprived of both mechanisms. Moreover, the recruitment of motor units, initially asynchronous, shifts toward a more synchronized pattern and the fatigued motor neurons require a higher excitatory input to ensure their firing rate. Finally, the firing rate of the motor units decreases [3,4,8]. 

At the peripheral level, electrophysiological adjustments consequent to fatigue onset include accumulation of both inorganic phosphate in the sarcoplasm and increase of intracellular pH. Imbalance of intra- and extra-cellular sodium and potassium concentration combines with impairment in calcium release and reuptake at the sarcoplasmic level and the inhibition of cross-bridges interactions [3,4]. As a result, altered neuromuscular transmission and action potential propagation occur [6,9]. These phenomena, combined with a changing strategy of motor unit recruitment, contribute to span the shape of the action potential, the electrical signal generated by all the motor units recruited by the central nervous system. A reduction of the conduction velocity, the speed at which the action potential propagates along the sarcolemma membrane, is attributed to fatigue onset and represents a focus point in the study of muscle contraction [1,4,6,9]. 

### 1.3. The Surface Electromyography

**Figure 1:**
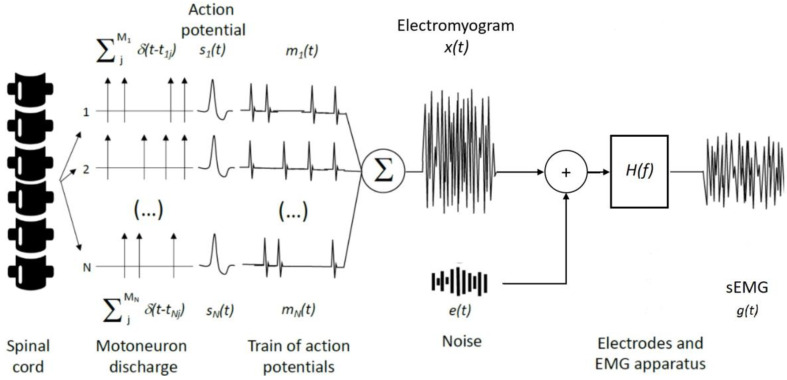
Schematic representation of the generation of electromyograms from motor unit action potentials. The recorded surface electromyography (sEMG) differs from the physiological electromyogram because of noise and filtering introduced by the detection; *g*(*t*) is the recorded signal on which spectral or complexity-based analyses are conducted, *x*(*t*) is the true signal of interest, based on neurophysiological backgrounds, *e*(*t*) is additive noise, and *H*(*f*) is the transfer function of the recording apparatus.

Muscle contraction is preceded by a cascade of electrophysiological events, from the excitation of motor neurons in the spinal cord to the propagation of action potentials across the muscle T-tubules. All these events, to a certain degree, contribute to the generation and propagation of electric potential in the surrounding tissues, referred to as electromyogram. The electromyogram is often termed as an interference signal, as it coalesces the contribution of many different motor units; depending on the contracting muscle and on the contraction intensity, the number of excited motor units may indeed range from tens to hundreds [10,11]. As schematically illustrated in Figure 1, the interference electromyography (EMG), x(t), may be modelled as the sum of trains of motor unit action potentials $m_{i}\left(t\right)$, each defined as the time convolution between the discharge instants $\delta \left(t-t_{ij}\right)$ and the waveform $s_{i}\left(t\right)$ of the action potential of each single unit:

(1) $$x\left(t\right)=\overset{N}{\underset{i}{\sum }}m_{i}\left(t\right)=\overset{N}{\underset{i}{\sum }}\overset{M_{i}}{\underset{j}{\sum }}\delta \left(t-t_{ij}\right)*s_{i}\left(t\right)$$

where N and $M_{i}$ respectively correspond to the number of motor units recruited and the total number of discharges ($j=1,2,3,\ldots ,M_{i}$) for the i-th motor unit. The degree of EMG interference is therefore clearly dependent on how often motor units discharge and on the number of motor units excited. Overtly, the degree of interference increases with the contraction level.

According to Equation (1), two main sources explain $m_{i}\left(t\right)$: the discharge instants *t_ij_* and the waveform representing the motor unit action potential, *s_i_*(*t*). Being the signal arising from the spinal cord and determining the onset and frequency of muscle excitation, the train of impulses characterising the motor unit discharge instants is regarded as the neural drive to the muscle [12]. The mathematical (Equation (1)) and conceptual (Figure 1) definitions for EMG do not necessarily imply a central origin for the discharge instants as often inappropriately conceived [9,13]; synaptic inputs arising from corticospinal pathways, spinal interneurons, and peripheral afferent feedback collectively determine the net neural drive to muscles [14]. Differently from the muscle neural drive, the waveform of motor unit action potentials does not carry any information from the spinal cord. It is entirely defined by peripheral factors, related to physiological, anatomical and detection aspects [15,16,17,18]. Physiological (e.g., conduction velocity, intracellular action potential duration) and anatomical (e.g., depth and length of muscle fibres) aspects are not under the direct control of the experimenter. On the other hand, detection aspects, as position and size of electrodes, should be cautiously defined according to the muscle studied and the purpose of the study. Considering t he widespread sampling of sEMG with a couple of surface electrodes, i.e., bipolar electrodes, here we therefore focus attention on the effect of bipolar montages. The magnitude of the bipolar montage transfer function may be approached as [19]:

(2) $$\left|H\left(f\right)\right|\propto \sin^{2}\left(\pi fd\right)$$

with d being the centre-to-centre distance between electrodes. Because of its high-pass filtering response for spatial frequencies smaller than 1/2d, the bipolar montage is a simple procedure for attenuating common mode signals associated with power line interference and far-field potentials [20,21,22]. Benefits of attenuation of the latter factor are well conceived in studies aimed at estimating conduction velocity [23] but may be questionable when the intention is to estimate force from EMGs [24]. Clearly from Equation (2), |H(f)|=0 at the frequencies f=n/d, n∈ℕ. Considering the multiplicative effect of H(f) on the EMG spectrum), the bipolar montage leads therefore to dips in the frequency spectrum G(f) of the recorded EMG [17,25].

The electrode filter function H(f) is particularly relevant when bipolar electrodes are aligned parallel to the underlying muscle fibres, whereby space and time are intertwined. In this case, the argument of the sine function in Equation (2) can be rewritten as πfd/v, with v corresponding to the action potential conduction velocity. This relationship between d and v could motivate attempts to define the appropriate inter-electrode distance not leading to spectral dips and methods for the estimation of conduction velocity from dips location in G(f) [26,27]. Both possibilities are arguable though, given they are valid for the specific case electrodes and fibres reside in parallel directions and because the conduction velocity differs between motor units. Moreover, the definition of appropriate inter-electrode distance in bipolar recording should not be based on the avoidance of spectral dips and of spatial aliasing [28] but on whether and how much both affect the possibility of extracting physiologically relevant information from the electromyogram. Although short distances may help attenuating the detection of undesired sources, non-targeted muscles, it may result in the detection of signals unrepresentative of the whole, target muscles. Notwithstanding the selectivity-specificity issue has been traditionally acknowledged [29,30], reports on this matter are incipient [31,32]. Throughout this review, we assume the bipolar EMG is both selective and specific, sampling exclusively from all fibres of the target muscle.

### 1.4. Surface EMG Analysis in Time and Frequency Domains

Different indices have been proposed to characterize the surface EMG (sEMG) in both time and frequency domains. Here we refer to these indices as sEMG descriptors. Time descriptors often convey information related to the amplitude of sEMG (i.e., amplitude descriptors) whereas spectral descriptors typically relate to the distribution of energy across the sEMG frequency or power spectrum. Restating the repertoire of time and spectral descriptors so far proposed appears pointless given recent reviews on this issue [33,34,35]. Our focus is rather on the most widely used descriptors and on their sensitivity to physiological, anatomical and detection aspects.

The sEMG can be conceived as a Gaussian random process with limited bandwidth [36]. The presence of random components in the signal makes unsuitable the use of specific waveform features, such as the peak or peak-to-peak value, to describe the amplitude of sEMGs. The sEMG amplitude is therefore more appropriately defined in statistical terms. Let’s consider the measured sEMG as a zero mean signal g(t) conveying trains of action potentials of different motor units, uncorrelated between themselves (Figure 1), and let’s call the power of individual trains of action potentials of each (i) of the N excited motor units, represented in time domain as $\sigma_{m_{i}}^{2}$ or in frequency domain as $P_{M_{i}}$, and the discharge rate and energy of the action potential of each motor unit as $DR_{i}$ and $E_{i}$ respectively. Then, the following relationships holds for the standard deviation or root mean square value of g(t) [12]:

(3) $$\sigma_{g}=\sqrt{\frac{1}{T}\overset{T}{\underset{0}{\int }}g^{2}\left(t\right)}=\sqrt{\overset{N}{\underset{i=1}{\sum }}\sigma_{m_{i}}^{2}\left(t\right)}=\sqrt{\overset{N}{\underset{i=1}{\sum }}P_{M_{i}}\left(f\right)}=\sqrt{\overset{N}{\underset{i=1}{\sum }}DR_{i}E_{i}}$$

where T corresponds to the period over which the sEMG has been recorded. According to Equation (3), the variance (power) of the recorded signal equals the sum of the power of individual trains of action potentials. Note that the additive property does not hold for the standard deviations: $\sigma_{g}\leq \sum \sigma_{m_{i}}$. Another interesting aspect in Equation (3) is the monotonic relationship between $\sigma_{g}$ and the discharge rate $DR_{i}$ and the energy $E_{i}$ of the action potential of each motor unit. These two aspects lead to considerations of practical relevance. First, owing to the temporal overlapping of positive and negative phases of excited motor units, an issue known as amplitude cancellation [37], not all motor units contribute to $\sigma_{g}$. Keenan et al [38] have shown however that normalization of $\sigma_{g}$ with respect to amplitude values obtained during a reference condition (e.g., maximal voluntary contraction) helps contending with the cancellation issue. Second, even though $\sigma_{g}$ is sensitive to both discharge rate and number of unit excited, it is also sensitive to any factors affecting the shape, and thus $E_{i}$, of motor unit action potentials, be them of physiological origin or not. The impossibility of distinguishing the contribution of both origins demands caution when drawing inferences from $\sigma_{g}$ [39], in particular when physiological and non-physiological factors may change abruptly and unpredictably like during dynamic contractions [40].

Different descriptors have been also proposed to characterize the EMG spectrum [13,18,41]. The most widely considered are the mean frequency (MNF) and the median frequency (MDF) defined as:

(4) $$MNF=\frac{\int_{f_{min}}^{f_{max}}f{\left|G\left(f\right)\right|}^{2}}{\int_{f_{min}}^{f_{max}}{\left|G\left(f\right)\right|}^{2}}$$

(5) $$\int_{f_{min}}^{MDF}{\left|G\left(f\right)\right|}^{2}=\int_{MDF}^{f_{max}}{\left|G\left(f\right)\right|}^{2}$$

with $f_{min}$ and $f_{max}$ defining the EMG bandwidth (typically ranging from 20 to 400 Hz). MDF is less sensible to noise [41] and more sensitive to simulated variations in the EMG spectrum [42] than MNF. Theoretical and experimental considerations upon the effect of discharge instants on the EMG spectrum revealed the rate of discharge of motor units (delta function in Equation (1)) contributes equally to frequencies over 30–40 Hz [18,43,44]. Consequently, and differently from its amplitude, the EMG spectrum is mostly dependent on the waveform of action potentials and not on the discharge rate of motor units. Factors affecting the waveform of action potentials may either change or scale its shape, as the filtering effect of the tissue interposed between electrodes and the excited fibers and the muscle fiber conduction velocity [16,45]. As for amplitude descriptors, the possibility of discerning the relative contribution of physiological, anatomical and detection source affecting spectral descriptors demands careful reflection.

Before commenting on the use and validity of amplitude and spectral descriptors during fatiguing conditions, a general consideration is necessary on EMG stationarity. The above descriptors presume the recorded EMG is stationary, at least in the wide-sense. Wide-sense stationarity is well accepted in applications for which variations in contraction intensity and in muscle shape and properties may be regarded marginal. These circumstances are often limited to laboratory applications, whereby isometric, constant force contractions may be applied. Even so, during such a controlled condition, non-stationarities may manifest, often related to the building up of muscle fatigue. On this regard, Bonato et al [42] wisely classified the sources of non-stationarities in sEMG as being either slow or fast. Slow non-stationarities are mostly associated with sluggish events, as the accumulation of metabolites in the muscle tissue or changes in temperature. Fast non-stationarities are related to any abrupt changes that could be triggered, e.g., by sudden variations in contraction level or in muscle length, both typically occurring in dynamic contractions. The effect of both non-stationarities may be circumvented by appropriately dimensioning the window over which spectral descriptors in isometric contractions are computed [41,46] or by averaging spectral descriptors across a few cycles, if possible, during dynamic conditions [42]. The crucial point though is not the non-stationarity itself but whether EMG descriptors are sufficiently sensitive and robust to detect physiological changes induced by the process under study and nothing else, be it fatigue or any other matter of applied relevance.

### 1.5. Myoelectric Manifestation of Muscle Fatigue in Time and Frequency Domains

Experienced sEMG users may wisely contest the potential of the technique to assess muscle fatigue. As defined here, and in agreement with others [9,14,18,47], muscle fatigue may be well assessed by any measurements of performance directly related to the reversible reduction of muscle force. Even the eye of an expert observer could accurately judge the onset of muscle fatigue. In these terms, the use of sEMG finds limited, if any, relevance. It is then that distinction between muscle fatigue and electrophysiological events leading to muscle fatigue must be distinguished. This discrepancy is well discussed in the classical review by De Luca [18]. The failure point, defining the onset of muscle fatigue and thus of a relevant reduction in force, power or performance in general, is preceded by alterations in the chain of events leading to voluntary contraction. These alterations, summarized in the illuminating work of Kirkendal [47], are hardly observable to the naked eye or to performance-measuring sensors. However, these alterations affect the electric potential generated in the surrounding tissues during muscle contraction, making of the sEMG a valid and popular means for studying signs of muscle fatigue. That is, the MMF [18,48,49]. The crucial point though is determining which sEMG descriptors are specifically sensitive to which of the physiological alterations most likely leading to muscle fatigue.

Both amplitude and spectral descriptors have been considered to assess MMF during fatiguing conditions. It is well established indeed that when performance is maintained at a constant level, before the failure point, the amplitude and the frequency spectrum of sEMG change [48,49,50,51]. The value of sEMG amplitude and spectral descriptors in studying MMF is however dissimilar, with spectral descriptors typically exhibiting more consistent variations during fatiguing contractions than amplitude descriptors. Multiple factors may account for this. When the sEMG is detected from muscles in which the fibers are aligned parallel to the electrodes, for example, the location of spectral dips depends on the conduction velocity ($f_{dip}=nv/d$; Section 1.3). In this circumstance, although the decrease in conduction velocity often reported in fatiguing contractions increases the energy of low frequency components, it also shifts the spectral dips to lower frequencies; both effects may therefore cancel out, not altering the total signal power and thus signal amplitude (see Figure 8 in [18]). Similarly, the decreased EMG amplitude expected for when the discharge rate of fatigued motor units decreases (Equation (3)) may be cancelled by the recruitment of additional, fresh units [52] (see Figure 8 in [14]). Motor unit recruitment is another—and possibly the most crucial confounding—factor affecting EMG amplitude. Motor units are known to have different sizes, with bigger units exhibiting a greater number of muscle fibers and thus greater action potentials. Even though one may argue the contribution provided by the recruitment of a big unit may outweigh that resulting from the recruitment of a small unit, the effect on EMG amplitude depends on the average distance of fibers of each unit to the electrodes (see Figure 2 in [39]). This issue is further aggravated if evidence on the rotation of motor units during fatiguing contractions is taken into consideration. Within a single muscle, different motor units have been shown to (rotate) be alternately recruited and de-recruited during prolonged, constant-force contractions [51,53,54]. If sEMGs recorded from a single muscle location do not convey information from the whole muscle [32], motor unit rotation may lead to decreases in sEMG amplitude and inferences on decreased excitation due to fatigue could be incorrect (see Figure 1 in [53]). Given all these competing factors cannot be controlled for, at least not during voluntary fatiguing contractions, amplitude descriptors may change unpredictably and their use to assess MMF may be unsuitable.

Physiological and non-physiological factors manifesting during fatiguing contractions are known to affect not just amplitude but also the spectral, sEMG descriptors. Differently, though, before the failure point is observed during a constant-performance condition, changes in MNF and MDF consistently indicate a relative shift in energy from high to low frequencies [15,18,41,42,46,48,50,55,56]. Such spectral compression is often attributable to decreases in conduction velocity with fatigue, possibly triggered by altered distribution of H^+^ and K^+^ across the sarcolemma [47]. The altered membrane excitability with fatigue may also lead to increased duration of intracellular action potentials, similarly leading to spectral compression [15]. The popular use of EMG spectral descriptors to study MMF is therefore presumably attributable to the fact they are equivalently affected by the different culprits of fatigue. The key question is which of these spectral descriptors is mostly sensitive and robust to describe MMF. Different indices have been proposed to characterize the spectral changes taking place with fatigue in the sEMG, based on different, time-frequency distribution approaches [13,55,57,58,59]. These studies have however devoted to much attention to comparing changes between traditional (MNF, MDF) and the proposed spectral indices without apparently caring for the validity of these changes. All these indices may indeed be flawed as none of these studies has controlled for actual variations in EMG spectrum. Comparing the performance of different indices from experimental data only seems unwise given the relative contribution of physiological and non-physiological sources arising in fatiguing conditions may be unpredictable. Rigorous, simulation studies have been published on this matter though [41,42,46,56]. From synthesized signals, for example, Bonato et al [42] observed that MDF computed from the Choi–Williams time-frequency distribution was shown to most accurately and robustly track abrupt and slow changes in the EMG spectrum typically occurring during dynamic contractions. The ability of MDF to capture the simulated changes was strictly related to focusing analysis on the most biomechanically repeatable portion of the cycle and to the averaging of the spectral descriptors over a few consecutive cycles; i.e., assessing MMF in dynamic conditions demands the underlying movement is repeated as consistently as possible until endurance. Collectively, these results indicate the traditional spectral descriptors may be well suited to study MMF during both isometric and dynamic condition, when certain methodological precautions are taken. EMG users must however be careful when inferences are to be drawn on the mechanisms underpinning fatigue from these spectral descriptors, as different mechanisms may affect them equally.

The considerations just presented for the EMG descriptors traditionally used to assess MMF apply likewise to any other proposed descriptors, many of which are illustrated in the next section. The validity of these indices may be acceptable only after they have been evaluated for robustness and sensitivity, during well-controlled, experimental and simulation conditions.

### 1.6. Myoelectric Manifestation of Muscle Fatigue in the Complexity Domain

The complex patterns of sEMG could be attributed to the mechanisms underlying its generation, which seem to be non-linear or even chaotic in nature, as it reproduces the non-linear electrical activity of the neuromuscular system [60]. In addition, the complex properties of sEMG seem to change with fibers contraction during muscle activation [61], potentially giving additional means to the linear sEMG analysis methods in assessing MMF [62]. Therefore, many different methods belonging to the classic non-linear time series analysis of biological signals have been proposed so far to obtain information on fatigue-induced adaptations of neuromuscular processes that could go unnoticed by linear analysis approaches [63]. The hypothesis is that, compared to linear and spectral indices, complexity measurements may detect additional EMG changes occurring with MMF. In the following of this review, readers will find the state of the art about complexity analysis applied to EMG signals, their qualities and the pitfalls that are settled in the procedures [64,65]. Awareness on the limitations of complexity-based methods will be also provided.

## 2. Materials and Methods

The measures of complexity of biological signals refer to the predictability of a time-series independently from the amplitude of its fluctuations [66], quantify its temporal irregularity [67] or its long-range (fractal) correlations [68] and estimate the amount of chaos in the underlying system [69]. To address all these aspects of complexity analysis, this review is based on the literature search of the PubMed and Scopus scientific databases using the following terms: EMG, fatigue, nonlinear analysis, complexity, fractal, nonlinear dynamic, entropy, approximate entropy (ApEn), sample entropy (SampEn), fuzzy entropy (FuzzyEn), multiscale entropy (MSE), recurrence plot analysis, detrended fluctuation analysis (DFA), largest Lyapunov exponent (LLE), correlation dimension (CD). Initially, a list of 333 articles was obtained. After having excluded duplicates papers and manuscripts dealing with pattern recognition and EMG classification, a subgroup of 109 studies was considered for the final analysis. Then, the review was limited to the 106 papers written in English without applying any other exclusion criteria. The collected papers were classified into four methodological groups: (1) fractals and self-similarity; (2) correlation; (3) entropy; and (4) deterministic chaos. For each method, we described its mathematical implementation and the influence of muscle activation and fatigue on the complexity indices. The physiological interpretation of the sEMG changes with muscle contraction, when available, aims at providing the reader a key to interpret the results when fatiguing contractions are investigated.

## 3. Results

### 3.1. Fractals and Self-Similarity

#### 3.1.1. Fractal Dimension

In 1977 Mandelbrot coined the term “fractal” to describe geometric shapes that reveal more details at increasing degree of magnification [70]. Three related features are accredited to fractal forms: heterogeneity, self-similarity and the absence of a well-defined scale of length. Heterogeneity reflects the property of showing emerging details the more closely the shape is examined. Self-similarity defines the characteristics of resembling similar structures at different size scale [68]. The description of fractal structures goes through the determination of fractal dimension (FD), an index characterizing “the complexity and space filling propensity of a structure” [71]. Transposed to time series signals, FD has been demonstrated to describe the self-similarity of a pattern over multiple time-scale [71,72]. FD can be estimated with different algorithms and a popular one is the Katz’s method [73] which, however, provides FD estimates that may depend on the length of the time series [74]. The Katz’s method has been revised by Anmuth et al. [61] to be applied to sEMG signal during isometric contractions. Given a signal lasting 3 seconds, FD was estimated for the middle 1 s as:

(6) $$FD=\frac{\log N}{\left[\log N+\log\left(\frac{d}{L}\right)\right]}$$

where *N* is the number of samples in the signal, *d* is the planar extent of the waveform (computed as the distance between the first point of the sequence and the point of the series that provides the farthest distance), and *L* the total length of the signal (sum of distances between successive points) [61,73]. 

Another popular FD estimator is the box-counting method. This algorithm superimposes the time series waveform with a regular grid of square boxes. The size (*S*) of the boxes is increased from small to large dimensions and the number (*N*) of boxes crossed by the waveform is computed for each size. FD is thus estimated as:

(7) $$FD=\frac{\log N}{\log\frac{1}{S}}$$

Since the fractals structures show an inverse power law relationship between *N* and *S*, *FD* in Equation (7) corresponds to the slope of the linear relationship between log*N* and log1/*S* [71]. In the study of Gitter, box sizes were chosen as a multiple of the -amplifier bit resolution and the sampling rate and its range varied from 2 to 500 boxes [71] (where a unit box had a physical dimension of 5580 μV/μs). FD values close to 1 reflect smoothed signals whereas values approaching 2 are typical of signals with high space-filling propensity [75]. The box-counting algorithm has been used to evaluate sEMG signals during isometric and isotonic contractions [76,77,78]. 

**Figure 2:**
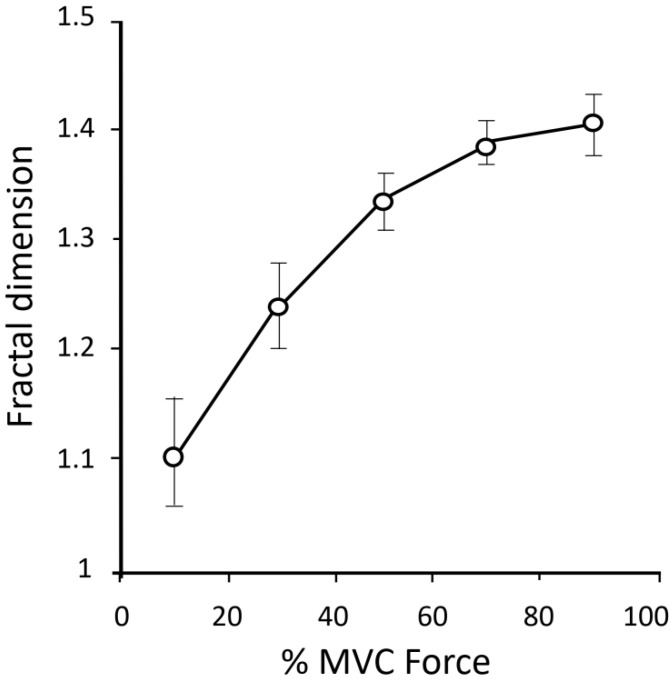
Averaged fractal dimension (FD) as a fraction of maximal voluntary contraction (MVC) force (redrawn from Gitter and Czerniecki, [71], with permission).

*FD and muscle activation.* Anmuth et al. [61], and Gitter and Czerniecki [71] investigated the behavior of FD as a function of force and found that, similarly to other traditional EMG indices, the average FD increased almost linearly with the force intensity for force values below 50% of the maximal force (Figure 2). Conversely, above this level the FD rise declined, deviating from the linear increase [61,71,79]. Similarly, Beretta-Piccoli et al. [80] found a low dependence of FD on force intensity. Indeed, they observed a linear relationship between FD and the level of force from 10% till 30% of the maximum voluntary contraction (MVC), but at higher force intensity FD leveled to a plateau. Even though these results led the authors to speculate the FD descriptor is “a reliable indicator of motor unit synchronization, less dependent from the firing rate” no direct evidence appears to confirm the sensitivity of FD to motor unit synchronization.

Xu et al. [79] determined FD on simulated EMG signals in which motor unit recruitment and firing rate was varied. They found that FD increased with the recruitment but the rate of the increment tended to plateau when recruitment was high. Moreover, firing rate influenced FD, but only for low values of recruitment [79]. 

Noticeably, not all the investigators found linear correlations between FD and force, neither at low level of force. Indeed, Troiano et al. [81] did not found any relationship between FD and percentage of MVC force in trapezius muscle, and similar results were obtained by Poosapadi and Kumar [82].

*FD and fatigue:* FD has been proposed to monitor changes in EMG signal as a consequence of fatiguing contractions [75,77,78]. Beretta-Piccoli et al. [75] used FD to investigate MMF in knee-extensors muscles, reporting the time-course of FD values in vastus lateralis and vastus medialis muscles during sustained contractions at different intensities. Analyzing the time course of FD during the development of fatigue a clear significant negative slope appeared, although different in the two muscles. The authors, citing the study of Mesin et al [78] in which a decline in FD was associated with a progressive MU synchronization, ascribed this behavior to an increase in MU synchronization as expression of the central nervous system adaptation to fatigue progression. Moreover, the investigators attributed the different slopes found between the two muscle bellies to the different proportion of slow and fast twitch fibers constituting the muscles. 

**Figure 3:**
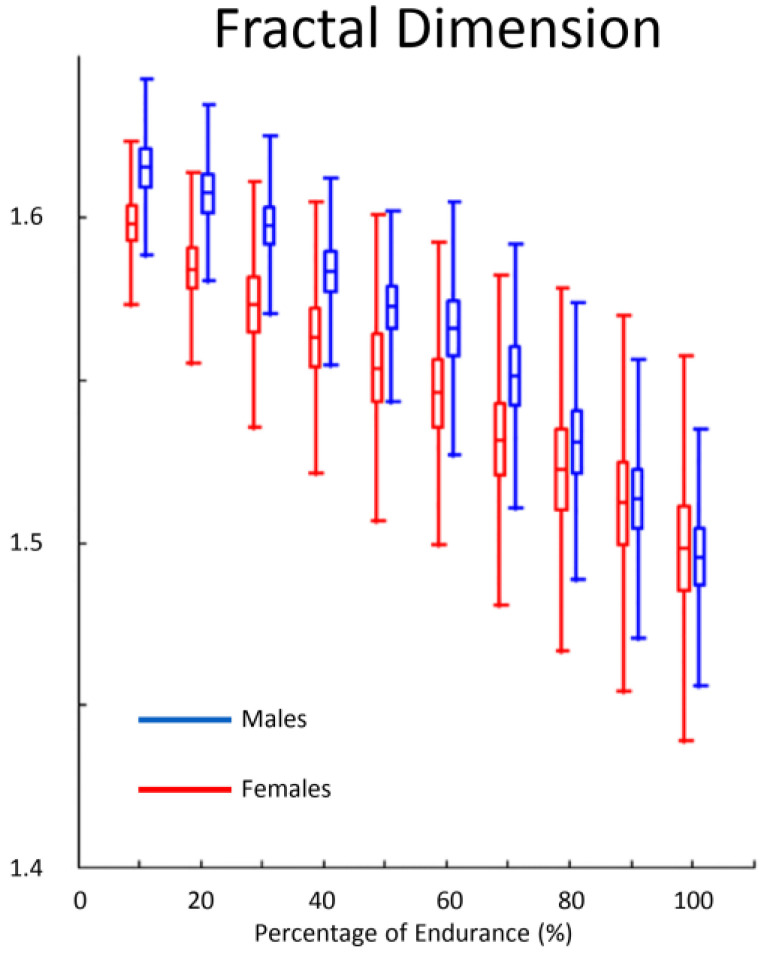
Mean percentage of changes in FD versus time in males (blue) and females (red) during 60% MVC prolonged contraction. The time scale is expressed as a percentage of the total exhaustion time for each subject (from Meduri et al., [85] with permission).

The decay of FD during sustained isometric contractions is the common denominator of the studies of Mesin et al. [78], Beretta-Piccoli et al. [75,83], Troiano et al. [81] and Boccia et al. [77]. Indeed, they found a linear decrease of FD during fatiguing contractions and attributed this response to an increase in motor unit synchronization (Figure 3). In [78], FD values showed no association with motor unit conduction velocity, supporting the idea that FD is more sensible to central rather than peripheral fatigue. Despite this, the authors drew these conclusions using advanced signal analysis techniques, the interference nature of the EMG signal makes questionable any speculation on the origin of the fatigue components (central rather than peripheral). 

Lin et al. [84] investigated the FD during isotonic repeated submaximal contractions (pedaling) but observed no change. Meduri et al. [85] also tested the existence of different gender-related resistance to fatigue in biceps brachii muscle. The time courses of conduction velocity and FD were determined during the time-to-exhaustion task. Investigators found a lower initial FD in females compared to males. Moreover, the rate of FD decrease at low level of contraction intensity was not different between genders whereas males showed a significantly higher decrement of FD during 60% MVC exhausting contraction. Importantly, the authors speculated the initial values of FD seem to be affected by motor unit synchronization as well as by subject fat layers and skin properties (Figure 3).

Troiano et al. [81] investigated the behavior of FD during fatiguing contraction at 50% MVC and found a significant fatigue influence on FD. Indeed, the rate of changes of FD determined during fatiguing tasks strongly correlated with endurance time, making this parameter a valuable tool to predict the time to exhaustion during an isometric task. 

Finally, Mesin et al. [86] explored the influence on FD on both different percentages of motor unit synchronization (from 0–20%) and different motor units firing rates (5–40 Hz). As previously anticipated, the Authors evidenced the existence of an inverse relationship between FD and motor unit synchronization and the positive relation with motor units firing rate. These findings have shed new light on the interpretation of fatigue-induced changes of FD, making FD no more considered as an exclusive index of motor unit synchronization [86]. 

Table 1 summarizes methodological aspects and results of studies on FD and muscle fatigue.

#### 3.1.2. Detrended Fluctuation Analysis.

A process g(*k*) is “self-similar” when it holds the same statistical properties of *a*^−H^*g*(*ak*), with H the Hurst exponent. This means that subsets of the original series properly rescaled to the size of the original one look statistically similar to the original, a property called "self-similarity". The Detrended fluctuation analysis (DFA) is a complexity method to assess the scaling properties of self-similar signals. The algorithm returns a scale parameter α which is strictly related to the Hurst exponent, with α = 0.5 in case of no correlation (white noise), α = 1 in case of “1/f” (or pink) noise, and α = 1.5 in case of Brownian motion (or random walk). In particular, 0 < α < 0.5 indicates anti-correlation between samples whereas α > 0.5 indicates long-range correlation [87]. 

To estimate α of a series *g*(*k*) of *N* samples, first *y*(*k*), cumulative sum of *g*(*k*), is calculated. Then:

1. *y*(*k*) is split into *M* non-overlapped boxes of size *n* (in general, *N* is not a multiple of *n* and thus the *M* boxes cover a segment *N*′ = *M* × *n* slightly shorter than *N*);
2. The local trend, *y_n_*(*k*), is determined in each box of size *n* by a least-squared linear detrending;
3. The difference between *y*(*k*) and the local trend is computed;
4. A variability function *F*(*n*) is calculated as the root-mean-square of the variance of the residuals in each box:
  (8) $$F\left(n\right)=\sqrt{\frac{1}{N^{\prime }}\overset{N^{\prime }}{\underset{k=1}{\sum }}{\left[y\left(k\right)-y_{n}\left(k\right)\right]}^{2}}$$
  The Steps 1–4 are repeated for different box sizes *n* and α is estimated as the slope of the regression line fitting *F*(*n*) vs. *n* in a log-log plot [87]. Successive improvements of the DFA method considered least-square detrending polynomials of order greater than one and were able to employ the whole series of *N* samples for each block *n* with properly overlapped boxes [88,89]. The popularity of the DFA method lies in the fact that unlike other estimators of the Hurst exponent it does not require to know in advance whether the fractal series belongs to the family of the fractional-Gaussian noises (fGn) or Brownian motions (fBm) [65]. The DFA provides acceptable estimates of H for both these classes, being α = H for an fGn process, and α = H + 1 for an fBm process [90].

*DFA and muscle activation*: Different studies demonstrated an increase of the DFA scaling exponent with muscle effort [91,92,93]. In addition, concentric contractions result in lower α values compared to isometric and eccentric contractions, with scale exponents close to one (the characteristic value of 1/*f* power law phenomena) suggesting a higher level of complexity [93]. Such difference were explained by the different levels of motor unit recruitment which occur during concentric versus eccentric contraction [94,95] and possibly by the different motor control strategy which regulates concentric and isometric contraction [96,97]. 

*DFA and fatigue*. The MMF as assessed by the DFA scale exponent results in a significant loss of signal complexity. Interestingly, Hernandez et al. [93] recently found a significant multivariate effect from fatigue status and muscle contraction type. They found that α DFA was significantly lower during non-fatigued compared to fatigued conditions and during concentric compared to isometric contractions. During fatigued condition, α was close to 1.5, value characteristics of Brownian motion. 

#### 3.1.3. Multifractality

Complex systems may also generate multifractal time series. A multifractal series is composed by interwoven fractal processes and specific methods of analysis should be applied to identify the components of the multifractal dynamics.

One multifractal method used in sEMG analysis is based on the evaluation of the singularity spectrum over successive epochs of 1s duration [62]. The measured signal is covered with boxes of size *l* and the probability *P_i_*(*l*) in each box *i* is calculated. For monofractal series, *P_i_*(*l*) increases as the power α*_i_* of the size *l*, the exponent α*_i_* being called the singularity strength. 

For the multifractal analysis, a normalized *P_i_*(*l*) measure is used:

(9) $$\mu_{i}\left(q,l\right)=\frac{{\left[P_{i}\left(l\right)\right]}^{q}}{\sum_{j}{\left[P_{i}\left(l\right)\right]}^{q}}.$$

The exponent *q* allows highlighting the different components of the multifractal time series. The normalized measure in fact amplifies the fractal components with greater singularity when *q* > 1 and those with lower singularity when *q* < 1. In particular, if the series is monofractal the singularity strength does not change with *q*. Thus, averaging α*_i_* over all the boxes *i* one obtains the function α(*q*) that provides a measure of the degree of multifractality. The singularity α determines the Hausdorff fractal dimension *f* of the data and therefore, as α changes with *q*, also *f* changes with α. The function *f*(α) that describes the fractal dimension as a function of the singularity strength is called the singularity spectrum. 

Another way to assess the multifractality of a time series is to extend the DFA method, which was originally proposed for monofractal series. This is done modifying the definition of the variability function *F*(*n*) in Equation (8) and calculating a variability function *F_q_*(*n*) which depends on the moment order *q* as:

(10) $$\{\begin{array}{ll} F_{q}\left(n\right)={\left(\frac{1}{M}\overset{M}{\underset{k=1}{\sum }}{\left(\sigma_{n}^{2}\left(k\right)\right)}^{q/2}\right)}^{1/q} & for\;q\neq 0\\ F_{q}\left(n\right)=e^{\frac{1}{2M}\sum_{k=1}^{M}ln{\left(\sigma_{n}^{2}\left(k\right)\right)}^{q/2}} & for\;q=0 \end{array}$$

where *M* is the number of blocks of size *n* and $\sigma_{n}^{2}\left(k\right)$ is the variance of the residuals in each block [98]. When *q* = 2, *F_q_*(*n*) coincides with the “monofractal” variability function *F*(*n*). The multifractal variability function amplifies the fractal components with greater amplitude when *q* > 0 and those with lower amplitude when *q* < 0. At each moment order *q*, a multifractal DFA coefficient, α(*q*), is estimated as the slope of the regression line fitting *F_q_*(*n*) vs. *n* in a log-log scale. If α(*q*) depends on *q* the series is multifractal while monofractal series are characterized by constant α(*q*) functions.

*Multifractality in muscle action*. Li et al. applied the method of multifractal DFA to the cross-correlation function between force and sEMG [99]. The results show a strong statistical self-similarity in the correlation sequences between force and sEMG signals, with fractal characteristics similar to 1/f noise or fractional Brownian motion. The multifractal DFA has been applied to the biceps brachii contraction, and it was observed that the sEMG signal is mono- and multifractal in different time scales, with “several fractal-scaling breaks” [100]. 

*Multifractality and fatigue*. The singularity spectrum *f*(α) of the sEMG signal of the biceps brachii was estimated during isometric contractions and the area of the singularity spectrum was taken as a concise index of the degree of the sEMG multifractality [62]. The results demonstrated that the area of *f*(α) consistently increased during the static contraction suggesting the use of *f*(α) for assessing muscle fatigue.

The multifractal DFA approach was used also to evaluate whether the effects of fatigue on the EMG signal could be estimated with greater accuracy than that of conventional indices of EMG such as the MDF of the sEMG power spectrum [100]. The observed changes in Hurst exponent in the fatigued muscle may be due to a reduction in conduction velocity in muscles fibers and to the enlarged motor unit action potential, which may increase the long-range correlation in sEMG at small time scales.

### 3.2. Correlation

#### 3.2.1. Correlation Dimension

In 1996, Nieminem and Takala demonstrated that sEMG is better modeled as the output of a non-linear dynamic system rather than as a random stochastic signal [101], suggesting the use of non-linear analysis methods. Among the non-linear methods, the evaluation of the correlation dimension (CD) [102] has been used to classify the sEMG dynamics, both at rest and during light and fatiguing muscle contractions. CD is a measure of the amount of correlation contained in a signal connected to the fractal dimension. The CD estimation requires the calculation of the correlation integral C(*r*), which is the mean probability that the states of the dynamical systems at two different times are close, i.e., within a sphere of radius *r* in the space of the phases. Given a time series *g*(*k*), the phase space is reconstructed by the vectors *G*(*k*) = [*g*(*k*), *g*(*k* + τ), …, *g*(*k* + (*m* − *1*)τ)]^T^ with *m* the embedding dimension and τ a delay. The correlation integral is then estimated by the sum:

(11) $$C\left(r\right)=\frac{1}{N^{2}}\overset{N}{\underset{\begin{array}{c} i,j=1\\ i\neq j \end{array}}{\sum }}\Theta \left(r-||G\left(i\right)-G\left(j\right)||\right)$$

where *N* is the number of states, Θ the Heavyside function and ‖…‖ the Euclidean norm. If *g*(*k*) is the output of a complex system, when *N* increases and *r* decreases, *C*(*r*) tends to increase as a power of *r*, *C*(*r*)~*r^CD^*. Thus, CD, the correlation dimension of the system can be estimated as the slope of the straight line of best fit in the linear scaling range region in a plot of ln (*r*) versus ln *r*.

The algorithm requires a large amount of data to provide reliable estimates, a restraint in the analysis of sEMG. Furthermore, the estimates are unreliable for *m* greater than 14 (Nieminem and Takala, 1996) and the computational time increases exponentially with the number of samples (Bai-Lin, 1990). 

*Correlation dimension and muscle activation*. The studies on correlation dimension applied to sEMG firstly confirm the non-linear character of muscle electrical activity, which shows a structure different from a pure random noise [103]. Thus, during the dynamic muscle contraction, neuromuscular system has been demonstrated to “progressively changes from narrow band orderly recruitment pattern to a broadband chaotic pattern” [103]. 

As it concerns muscle activity, EMG signals from lower limbs muscles during walking were found to exhibit signs of chaotic behavior by the computation of CD values between two and three [104,105]. Furthermore, the study of the electrical activity of paravertebral muscles during different bending postures demonstrated that CD is a reliable method to compare the EMG signal in various muscle contraction conditions [106]. Finally, during a submaximal test of isometric loading Meigal et al. demonstrated that correlation dimension was able to distinguish the sEMG characteristics between two groups of young and old healthy individuals [107]. 

More recently, Wang et al. used a mixed mathematical approach, based on decomposing the sEMG signal by the wavelet transform for calculating CD, to distinguish four types of forearm movements. This could be prospectively useful to classify muscle movements in the conception and design of new powered limb prostheses [108].

*Correlation dimension and fatigue*. Muscle fatigue seems to reduce the dimensionality of the system, as assessed by CD: this has been ascribed to motor unit synchronization and reduction in action potential velocity and firing rate, which may reduce the neuro-muscular system adaptability [101]. However, a precise connection between the physiologic adaptation to fatigue in muscle activity and the changes in correlation dimension of sEMG signals is still lacking.

#### 3.2.2. Recurrence Quantification Analysis

The recurrence quantification analysis (RQA) is a nonlinear geometrical tool used “to bring out temporal correlations in a manner that is instantly apparent to the eye” [109]. This analysis was proposed by Eckmann in 1987 to detect recurring patterns and non-stationarities in a dynamic system [110]. Given a data set *x*(*i*) of points, RQA is constituted by a recurrence plot in which an array of dots is arranged in a square map and darkened pixels are plotted at specific coordinate *i*, *j* whenever the point *x*(*j*) is closer than a distance threshold *r* to the point *x*(*i*). When the distance between *x*(*i*) and *x*(*j*) is below *r*, *x*(*j*) is considered as recurrent and then, a dot is signed on the recurring map at the coordinate (*i*, *j*). Given a time series *g*(*i*) its recurrence plot is obtained as follows:

1. Setting an embedding dimension (*d*) and a delay *τ*, the data set *x*(*i*) = (*g*(*i*), *g*(*i* + *τ*), …, *g*(*i* +(*d* − 1)*τ*)) is generated;
2. The radius *r* is set to a value that allows a reasonable number of *x*(*j*) data being closer than *r* to *x*(*i*);
3. A darkened dot is plotted at each coordinate (*i*, *j*) for which *x*(*j*) is included in the ball with radius *r* centered at *x*(*i*).

Since *i* and *j* are times the resulting recurrence plot provides information on the time correlation of the data set. 

Different recurrent structures might be found looking at the recurrence plots [111,112]. Single isolated points result from chance recurrences in the signal; upward diagonal lines reflect the presence of a deterministic rule into the signal as they appear “whenever strings of vectors reoccur further down the dynamic” [113]; vertical and horizontal lines indicate the occurrence of isolated vectors of data set that match with a repeated string of vectors separated in time; and blank bands are the consequence of transients in time series. Given that subtle patterns are not always detected, different quantitative descriptors can be determined. Readers can found an exhaustive description of the recurrence plot descriptors in the brilliant paper of Webber and Zbilut [112]. The most often used are: 

(i) Percent determinism (%DET), that quantifies the percentage of recurrent points forming diagonal line structures

(12) $$\%DET=\frac{\sum_{l=l_{min}}^{N}lP\left(l\right)}{\sum_{i,j}^{N}R_{i,j}}$$

where *P*(*l*) is the frequency distribution (i.e., the probability) of diagonal lines with length *l*, being *l* an integer number;

(ii) Percent recurrence (%REC), that quantifies the density of recurrent points in the plot:

(13) $$\%REC=\frac{1}{N^{2}}\overset{N}{\underset{i,j=1}{\sum }}R_{i,j}$$

A critical aspect of RQA is the need to carefully tuning the embedding dimension, the delay *τ* and the threshold distance to obtain reliable estimates [78,111,114]. A typical value of the delay *τ* is the first zero of the autocorrelation function.

*RQA and muscle activation*. Several studies investigated the sensitivity of RQA to sEMG shifts towards more deterministic behaviors under different contraction intensities and characteristics in both small and large muscles [114,115,116,117,118]. Filligoi and Felici [113] evaluated %DET during voluntary contractions at three different force levels, each sustained for 20 seconds. Although the initial %DET value was insensitive to force levels, the slope correlated with the contraction intensity. 

RQA behavior was also investigated in response to different levels of motor unit synchronization by computing %DET before, during, and after the injection of a drug to increase the motor units synchronization [118]. %DET rose as a function of synchronization in most of the investigated muscles, leading the authors to consider it as a suitable tool to monitor changes in motor unit synchronization [118]. Different results were reported by Schmied et al. [119] that did not find a correlation between %DET and the amount of synchronous impulses when contractions were performed at a low level intensity. No correlations were also found between %DET and potentiation phenomena neither in endurance-trained nor in power-trained athletes [120]. 

Some studies compared recurrence analysis to frequency analysis finding prompter response and higher magnitude of %DET compared to spectral indices. This supports the idea that recurrence indices present a higher sensitivity than spectral indices to detect sEMG drifts [114,116,121].

*RQA and fatigue.* RQA also explored the effects of fatigue on muscular activation in different studies, which found a continuous rise of %DET as a function of time, although the results could be influenced by factors such as contraction intensity, muscle size [78,111,116,121,122,123,124], altitude and other muscles characteristics [122]. The role of contraction intensity was explored by Webber and Zbilut who found an almost-steady-state behavior of %DET during sustained light loading whereas during heavy loading a progressive rise occurred [111]. 

**Figure 4:**
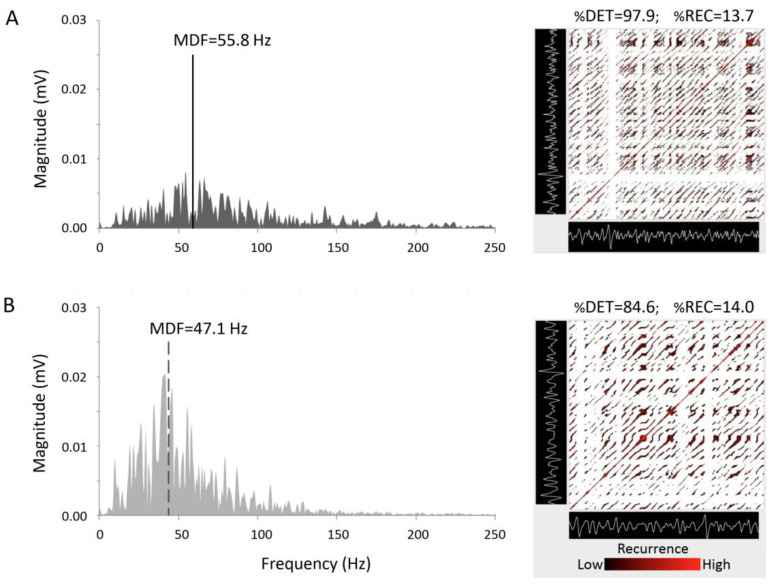
Power spectra with median frequency (MDF) (left panels) and recurrence plots with percent determinism (%DET) and percent recurrence (%REC) from recurrence quantification analysis (RQA, right panels) of sEMG signals for the non-fatigued (**A**) and fatigued (**B**) vastus lateralis muscle in one representative subject (personal data); analysis parameters are: *N* = 1024; *τ* = 4; *m* = 4; *r* = 15.

RQA was also adopted to characterize fatigue effects in different groups: power-trained athletes, endurance athletes, wheelchair basketball players and sedentary control subjects [116,120,122,125,126]. While %DET increased in all the athletes’ phenotypes, it did not in control group. Changes in %DET in athletes were ascribed to a more regular and more similar bursts pattern, while differences between groups were explained with the different proportion in fibers composition. Figure 4 shows an example, in a representative subject, of the computation of EMG power spectrum (with the calculation of the median spectral frequency) and RQA plot (personal data) during non-fatigued and fatigued muscle conditions. During fatigue, the computed mean spectral frequency decreases and the spectral power increases. Furthermore, the density of recurrent points remains relatively unchanged (constant %REC), but the arrangement of points is altered, indicating an increased periodic component in the EMG during fatigue.

Muscle endurance was also evaluated by RQA after exposure to high altitude. Similarly to normobaric condition, %DET progressively increased during the sustained contraction; however, the slope became steeper under exposure to hypobaric hypoxia [122]. Two studies evaluated the behavior of spectral variables and recurrence-plot indicators (%DET and %REC) on experimental as well as simulated EMG signals. In these latter the response to two typical signs of muscular fatigue, like reduction of conduction velocity and the increase in motor unit synchronization, were explored. %DET and %REC showed to be influenced by the conduction velocity and by the degree of synchronization [78,116]. Ito and Hotta, by the use of RQA, recently explored sEMG behavior during exhausting contraction under blood flow restriction. They found an increase in %DET during contraction and even higher values when blood flow restriction was applied [127]. Table 2 summarizes the parameters adopted for RQA analysis in previous studies and the results in terms of %DET and %REC in fresh and fatigued muscles. 

### 3.3. Entropy

#### 3.3.1. Approximate Entropy, Sample Entropy and Fuzzy Entropy

In 1991, Pincus coined the term approximate entropy (ApEn), to indicate a method estimating the “likelihood that runs of patterns that are similar remain similar on next incremental comparisons” [67]. An advantage of this method is its applicability in noisy and short datasets [128,129,130]. To calculate ApEn of a series *g*(*i*) of *N* equally-spaced values, one should first set an embedding dimension *m* and a distance threshold *r* and then:

1. Form a series of *N − m* + 1 vectors of *m* components *G*(*i*) = [*g*(*i*), *g*(*i* + 1), …, *g*(*i* + *m*)]^T^;
2. Compute the distance between any couple of vectors *G*(*i*) and *G*(*j*) as the largest absolute difference between the corresponding scalar components (if the difference is less than the distance *r* the two vectors are similar);
3. Count $n_{i}^{m}\left(r\right)$, number of the *N* − *m* + 1 vectors *G*(*j*) similar to *G*(*i*) and the probability to find a vector similar to *G*(*i*) as:
  (14) $$C_{i}^{m}\left(r\right)=\frac{n_{i}^{m}\left(r\right)}{N-m+1}$$
4. Calculate *C^m^*(*r*) as the average of $C_{i}^{m}\left(r\right)$ for all the vectors *G*(*i*);
5. Repeat the steps from 1 to 4 for the embedding dimension *m* + 1.

Then,

(15) $$ApEn\left(m,\;r\right)=-ln\left[\frac{C^{m+1}\left(r\right)}{C^{m}\left(r\right)}\right]$$

Deterministic sequences present a high degree of regularity, i.e., if they are similar for *m* points they are likely similar also for the next point, *m* + 1. Therefore, higher is the regularity, lower is ApEn. Since each sequence matches itself, ApEn is a biased estimator and it is lower than expected for short records [128]. This also implies that it lacks relative consistency, making it difficult to interpret the comparison of different datasets. Moreover, because of its bias, ApEn depends on the signal length. When two time-series are compared, care must be taken to estimate ApEn on the same signal durations [130].

Sample Entropy (SampEn) addresses the drawbacks caused by self-matching and provides better consistency and performance than ApEn [128]. SampEn reduces the bias avoiding self-comparison between vectors [130]. This is done by calculating $n_{i}^{m}\left(r\right)$, the number of vectors similar to *G*(*i*), for all the vectors *G*(*j*) excluding *j* = *i*. This leads to defining SampEn as:

(16) $$SampEn\left(m,\;r\right)=-ln\frac{A^{m}\left(r\right)}{B^{m}\left(r\right)}$$

where:

(17) $$A_{i}^{m}\left(r\right)=\frac{n_{i}^{m+1}\left(r\right)}{N-m-1}$$

(18) $$B_{i}^{m}\left(r\right)=\frac{n_{i}^{m}\left(r\right)}{N-m-1}$$

Boasting better consistency and robustness, the fuzzy approximate entropy (FuzzyEn) was proposed in 2010 for noisy and short datasets [129,131]. Additionally, FuzzyEn was independent of the tolerance *r* introducing the concept of fuzzy membership functions for determining the degree of similarity between patterns. Therefore, the similarity between *G*(*i*) and *G*(*j*) is quantified by a fuzzy continuous and convex function [129,132]:

(19) $$C_{i}^{m}\left(r\right)=\frac{1}{N-m+1}\overset{N-m+1}{\underset{j=1,\;j\neq \;i}{\sum }}\Omega \left(d_{i,j}^{m},r\right)$$

with [121,131,133,134]

(20) $$\Omega \left(d_{i,j}^{m},r\right)=e^{-\frac{d_{i,j}^{2}}{r}}$$

Finally,

(21) $$FuzzyEn\left(m,\;r\right)=-ln\left[\frac{C^{m+1}\left(r\right)}{C^{m}\left(r\right)}\right]$$

where *C^m^*(*r*) is the average of $C_{i}^{m}\left(r\right)$ for all the vectors *G*(*i*).

#### 3.3.2. Multiscale Entropy

The measures of entropy like SampEn cannot properly distinguish whether the irregularity of the time series just reflects random components or whether it is generated as the output of a genuine complex system. To better detect the presence of complexity in the time series some authors proposed a multiscale approach to entropy [135]. The multiscale entropy method is based on the evaluation of SampEn on progressively coarse-grained series. A coarse graining of order *n* consists in applying a moving average filter of order *n* on the original series *g*(*i*) and in decimating the filtered series taking one sample every *n*. Then, SampEn is estimated over the coarse-grained series obtaining the multiscale entropy at the scale *n*, MSE(*n*). Clearly, MSE (*n* = 1) coincides with SampEn by definition. Like SampEn, also the multiscale entropy needs the preliminary choice of the proper embedding dimension *m* and threshold distance *r*. In addition, it is still unclear whether the same threshold *r* should be used at all the scales *n* or whether it should be adjusted at each scale, *r*(*n*) [136]. Recently, the coarse-graining procedure has been improved to allow stable estimates at large scales even when analyzing relatively short data segments and to reduce leakage from the shorter to the larger scales due to the wide transition band of the moving average filter [137]. A concise way to quantify the MSE(*n*) profile is to sum all scales shorter than a critical scale *τ_c_* to obtain a short-term complexity index, *C_S_*, and to sum all the scales larger than *τ_c_* up to the largest estimated scale, *n_max_*, to obtain a long term complexity index, *C_L_*, as:

(22) $$C_{S}=\overset{\tau_{c}}{\underset{n=1}{\sum }}MSE\left(n\right)$$

(23) $$C_{L}=\overset{n_{max}}{\underset{n=\tau_{c}+1}{\sum }}MSE\left(n\right)$$

**Figure 5:**
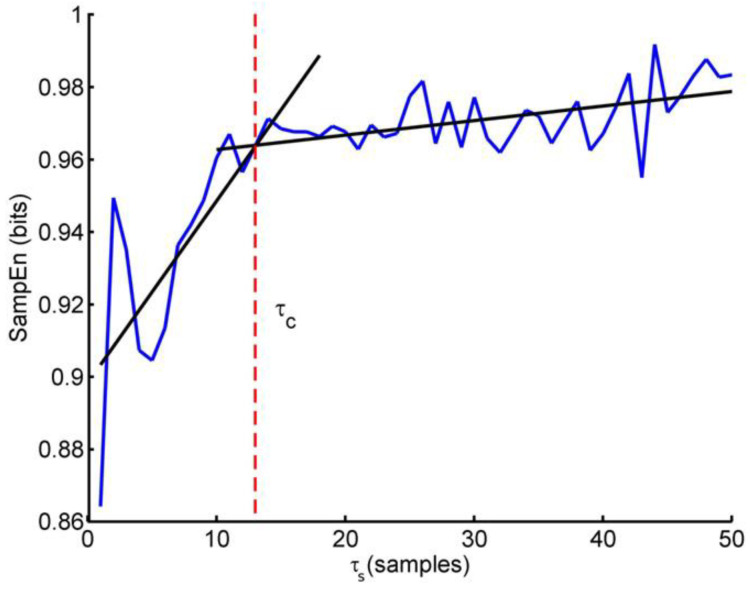
Example of sEMG multiscale entropy and identification of the critical scale τ_c_ for the definition of short-term and long-term complexity (from [138] with permission).

To identify the critical scale *τ_c_* analyzing sEMG during isometric contractions, Cashaback et al. performed a piecewise-linear regression on MSE(*n*) estimates for scales *n* between 1 and 50 samples (corresponding to the range between 0.004 and 0.2 s) and found a single breakpoint demarcating two linear scaling regions [138]. The intersection of the two-piece regression defined *τ_c_* (see Figure 5).

*Entropy and muscle activation*. Several studies used entropy-based methods in characterizing the complexity of EMG signals during relaxed conditions [139] and contractions [117,131,140]. From a physiological viewpoint, as healthy biological systems show markedly higher complexity than compromised ones, low entropy values could be read as a sign of impairment [141].

Despite the different studies using ApEn on EMG signals, its consistency and reliability have recently been questioned [72]. Zhou et al. employed SampEn and FuzzyEn to interpret sEMG collected at different intensity levels of contraction and found a very weak correlation between SampEn and muscle torque while FuzzyEn showed a direct positive correlation with the effort [134]. These authors concluded that FuzzyEn could be a useful alternative to force estimation whereas SampEn might be determined as a biomarker of EMG able to overcome interference due to changing muscular contractions intensity. A relationship between entropy measures and force production was also examined by Troiano et al., [81] who found no effect of fatigue on entropy values.

Finally, MSE analysis was applied to sEMG signal by Cashaback [138] to evaluate the short-term complexity of sEMG at three different intensity contractions. The authors reported a correlation between MSE and contraction intensity although the level of complexity at 100% was only slightly different compared to the one found at 70%. The investigators hypothesized that, given that force production above 70% is mainly attributed to an increase in temporal firing, signal complexity might be mainly influenced by rate discharge rather than motor unit recruitment [138].

**Figure 6:**
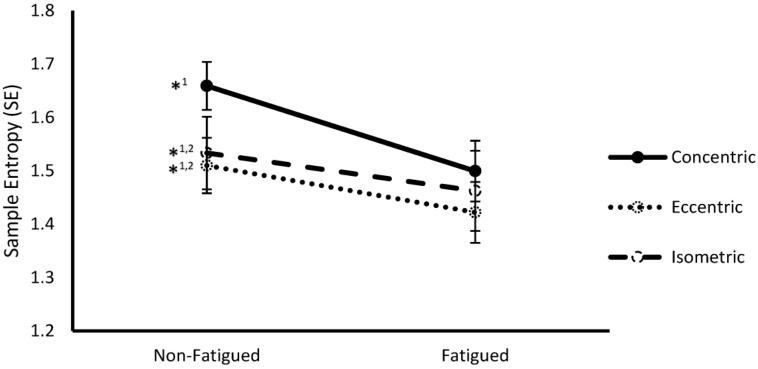
Sample entropy (mean ± SEM) of vastus lateralis sEMG signals from non-fatigued to fatigued conditions during concentric, eccentric, and isometric contractions (from Hernandez et al., [93] with permission).

*Entropy and fatigue*. The use of entropy algorithms to study MMF has been recently evaluated [121,129,132,142]. Hernandez et al. [93] recently studied the individual influence of fatiguing contractions and of different contraction types on the complexity of sEMG signal by SampEn and DFA. The effect of the combination of both factors were also evaluated. Given that SampEn values decreased in fatigued conditions and different values were found among the contraction types, the Authors concluded that “sEMG complexity is affected by fatigue status and contraction type, with the degree of fatigue-mediated loss of complexity dependent on the type of contraction used to elicit fatigue” [93] (Figure 6).

Lin et al. applied the SampEn algorithm to sEMG signals collected from quadriceps muscles during cycling. Comparing the results obtained under fatigued and un-fatigued conditions they found no differences in SampEn values [84]. The absence of any changes in signal EMG complexity was attributed to the different type of contraction (isometric and cyclic). 

FuzzyEn was also used to characterize the determinism of sEMG signal during fatigue [129,132,142]. The study of Xie et al. compared the time course of FuzzyEn with that of ApEn and of the MDF and found that FuzzyEn decreased linearly during muscle contraction as well as the MDF, where ApEn did not [129]. Successively the Authors compared the performance of FuzzyEn with SampEn and ApEn and concluded in favor of FuzzyEn, due to its better robustness to the analysis length [132]. Navaneethakrishna et al. [142] applied FuzzyEn to explore determinism in sEMG signal under fatigued and un-fatigued conditions and, similarly to previous studies, found a decline in entropy throughout fatigue development. 

Kahl and Hofmann [121] compared six different algorithms (including SampEn and FuzzyEn) in the detection of local MMF. The sEMG signal was analyzed by spectral, entropy and recurrence quantification analysis. Authors found that entropy-based variables performed better than recurrence methods, though ApEn provided a low MMF detection quality. Better results were found from SampEn. Moreover, a limit of FuzzyEn method was recognized on the high computational effort.

The above cited work of Cashaback et al. [138], based on MSE approach, found that entropy values significantly decreased after fatigue. The authors hypothesized that the reduction of signal complexity might have resulted from a decrease of action potential amplitude and velocity as a consequence of alterations in the metabolic and enzymatic events involved in muscle contractions. Similarly, Navaneethakrishna et al. [142] observed a clear reduction of MSE values with MMF and attributed the finding to the fatigue-induced synchronization of motor unit recruitment that in turn would have led to the generation of more regular pattern in the neuromuscular signal.

MSE was used to investigate MMF also in a group of children with cerebral palsy to have a deeper insight into the central nervous system and neuropathological mechanisms underpinning muscle contractions [143]. Investigators noticed a decreasing pattern of MSE along with fatigue development and ascribed it to a reduction of motor unit synchronization.

Table 3 shows settings and results obtained using entropy algorithms in the studies taken into considerations.

### 3.4. Deterministic Chaos

#### Largest Lyapunov Exponent

The determination of the chaotic properties of a nonlinear system may be performed through the computation of largest Lyapunov exponent (λ_LLE_), which estimate the rate of exponential divergence of neighboring trajectories into the phase space. This measure can therefore quantify the “amount of chaos” in a system. Different algorithms have been implemented to determine λ_LLE_ from finite amounts of experimental data. The first implementation by Wolf estimated the non-negative Lyapunov exponent and determined the grade of unpredictability by the magnitude of the exponent, but it was rather inefficient [144]; later, the Rosenstein’s method proved to be more efficient and overcame the drawbacks of the Wolf algorithm [145]. Rosenstein’s algorithm requires four input variables: time delay, minimum embedding dimension, mean period and maximum number of iterations. Briefly, the EMG time series of *N* points is considered as a trajectory in the embedding space. The algorithm locates the nearest neighbor of each point *j* of the trajectory, and considers the distance between these two close points as a small perturbation, Δ*_j_*(0). It is assumed that the *j*-th pair of nearest neighbors diverges in time at the exponential rate given by the largest Lyapunov exponent λ_LLE_, which means that *ln*Δ_j_(*i*) = C*_j_* + λ_LLE_*i*. This equation, evaluated λ_LLE_ for all the *j* pairs, represents a set of parallel lines. To reliably estimate λ_LLE_ from short and noisy data, the average of the parallel lines is computed. In general, the average line shows a long linear region after a short transition, and is estimated as the slope of the regression line fitting the average line. 

*Muscle activation andλ_LLE_*. The Rosenstein method for calculating λ_LLE_ was applied on EMG signals by Chakraborty and Parbat [72] for the assessment of chaotic patterns during isotonic contractions of biceps brachii muscle (arm flexion with 1 kg load). Considering the stochastic nature of EMG, the authors used Cao’s method for determining the embedding dimension [146], whereas the time delay was determined through Kraskov’s mutual information function [147], the mean period was obtained as the reciprocal of the median frequency found by the average Welch periodogram technique and 100 iterations were used as the last input variable. The results obtained by this application suggested the presence of deterministic chaos in EMG signal, and found an, although very limited, variability with the applied load. In another study, when applied to the electrical activity of paravertebral muscles during various bending postures, the positive Lyapunov exponent could not discriminate the contraction conditions, differently from CD [106].

*Muscle fatigue andλ_LLE_*. The estimation of the largest Lyapunov exponent had limited applications in the evaluation of muscle manifestation of fatigue. The λ_LLE_ value did not change with the increase of the muscle load in [72], although it was unlikely that the load used in this work provoked a significant fatigue state in the tested muscle (biceps brachii). Significant reductions in the dynamic stability of low back EMG were found during a fatiguing task (30 repetitions of trunk extension) by means of the maximum Lyapunov exponent [148]. Interestingly, the index was lower in subjects with chronic low back pain (in whom paravertebral muscles are often contracted for antalgic reasons) compared to control subjects, with a trend more pronounced in people with low back pain toward a reduction during asymmetric versus symmetric tasks [148]. In a work of Padmanabhan and Puthusserypady [104] sEMG signals exhibited chaotic behavior with a greater number of positive Lyapunov exponent for signals recorded during maximal voluntary contraction than during walking. Finally, Sbriccoli et al., [149] demonstrated a significant reduction (by 14–42%) of λ_LLE_ in EMG from muscles with exercise-induced muscle damage (by 35 maximal contractions of biceps brachii), with complete recovery after two weeks.

## 4. Discussion

**Figure 7:**
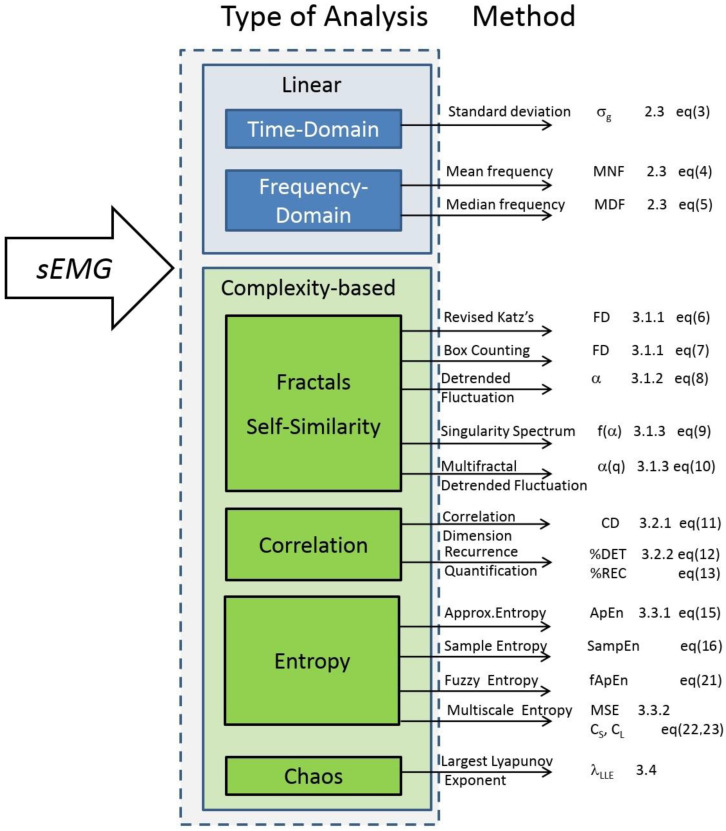
Scheme representing the considered linear and complexity-based indices for the sEMG analysis.

This review aimed at describing the main linear and complexity analysis methods in the literature which were applied to the EMG signal to determine the effects of fatigue on muscle electric activity (the scheme we followed is summarized in Figure 7). The issue we reviewed plays an important role in physiology (e.g., exercise physiology, neurophysiology, training, etc.) and pathophysiology settings (physical rehabilitation, neurology, prosthesis development, etc.). 

Some linear and spectral descriptors of EMG, as the σ_g_ and MDF have been demonstrated to be sensitive to fatigue-induced variations of EMG. However, intriguingly, it has been shown that the EMG signal also exhibits many complexity characteristics deserving to be evaluated, especially to understand whether these features have an onset time and a sensitivity to MMF development different from those of the classic linear descriptors. 

Several papers focusing on the complex behavior of EMG demonstrated so far that the EMG signal is non-linear in nature and expresses the features of a low dimension chaotic system [72,100,104]. Many complexity indices have been therefore used in characterizing the changes occurring in EMG with muscle activation and with fatigue. Some of them seems to be more informative and shows early changes compared to traditional linear and spectral analysis. In addition, fatigue results in a significant loss in EMG complexity [124,127,143,148]. 

Among the complexity analyses applied so far to the EMG the fractal analysis had many applications. Though not universally accepted [81,82], FD typically reveals an increase during muscle activation at low intensity levels of force production [61,76,79,82] with a decrease in response to MMF [64,65,66,79,84,86]. The common finding of the reported studies suggests an inverse relationship between FD and motor unit synchronization. By contrast, FD seems to be positively related to motor unit firing rate [78,86]. Finally, it showed to be suitable to estimate the exhausting time during an isometric contraction [80].

The RQA approach is another widely used index of complexity applied to EMG. A rise in %DET was attributed to an increase in motor unit synchrony and in a more similar bursts of motor unit potential action generation patterns. Local MMF is accompanied by an increase in recurrent statistics in sEMG signal therefore, %DET represents a promising tool in revealing early onset the MMF during a challenging motor task [45,78,111,121,122,123,124].

In addition, the entropy-based measurement has been widely used to evaluate how fatigue influences the determinism of EMG signal. During fatigue development entropy parameters show a clear decline, reflecting a shift of EMG towards more regular pattern. The decay observed in sEMG complexity by entropy has been ascribed to a decrease of both action potential amplitude and velocity probably due to alterations in metabolic and enzymatic events involved in muscle contractions [93,129,142,143].

A promising extension of MMF detection capabilities by complexity indices applied to EMG was introduced by the study of multifractality [62]. This method has shown a higher degree of correlation and accuracy with the progress of fatigue compared to the median spectral frequency, and presents possible applications, such as discrimination between normal and pathological sEMG (e.g., in those neuromuscular disease where a reduction of the number of motoneurons occurs and the action potential of the residual motor units changes in shape and duration) [100].

Finally, the determination of the largest Lyapunov exponent from sEMG demonstrated the chaotic properties of this nonlinear system but its potential in detecting MMF seems to be limited [72,106]. Therefore, despite some intriguing results [104,148,149], future standardized fatiguing protocols are needed to confirm whether λ_LLE_ of sEMG can be diagnostic tool to assess MMF and impairments, as well as the effectiveness of treatment in different settings, as clinic (rehabilitation) and sporting contexts.

All these findings, collectively, might make the use of complexity analysis tempting. However, readers have to consider the several pitfalls and tricks thronging the analysis process. Indeed, almost all the complexity procedures present some limitations in their use that should be considered. First, the quality of the estimates of complexity indices increases with the length of the dataset and for this reason complexity methods generally require long time series: this may be a critical point because EMG data during fatiguing muscle contractions are usually of reduced length. Therefore, there is a need to develop indices and estimation algorithms which can be meaningfully applied to short dataset. In this regard, recent lines of research in the complexity analysis of physiological signals are aimed at specifically designing algorithms for short time series, for instance by reducing the estimator bias and variance in multiscale entropy analysis [137,150,151] or by improving the consistency of multifractal DFA estimates [88]. It is, therefore, desirable that these algorithms are properly adapted to the analysis of sEMG and applied to detect the electromyographic manifestation of muscle fatigue.

Second, many of these analyses are based on highly recursive calculation procedures and therefore needs high computational times. Third, from a statistic viewpoint, there is a requirement for surrogate data analysis, in order to test the EMG signal for non-linearity in different conditions (e.g., fatigued vs. non-fatigued states). In the vast majority of the studies cited in this review no surrogate data analysis has been performed. Fourth, only in some cases an accurate parameterization of the variables used in the specific complexity analysis (in particular the parameters used to reconstruct the phase space, as the embedding dimension, the time delay, the critical scale and the threshold distance) has been performed. This latter point has been deeply stressed in those studies. Indeed, given that an inaccurate setting of the algorithms parameters severely impacts on final results, a meticulous detection of the most appropriate setting is absolutely required to achieve reliable results and avoid improper conclusions. We encourage the interested readers to undertake the endeavor of assessing the sensitivity of complexity descriptors with synthetic EMG signals, whereby the effect of different sources leading to MMF can be controlled for. It is our understanding that only then would it be possible to reveal the added value of complexity analysis in screening the various physiologic phenomena that may manifest in experimental EMG signal during fatiguing contractions (synchronization of the motor units generating the action potentials, changes in the shape of action potentials, in the firing rate, in the biochemical conditions and metabolism of the muscle fiber, etc.). Indeed, in some analyses reported in this review, the authors attempted to correlate the behavior of the complexity indices of EMG to the changes in the physiological phenomena that underlie the MMF during a protracted muscle contraction. However, this should be possible only when working with synthetic signals, in which several phenomena, such as fiber recruitment and action potential synchronization, can be controlled. Differently, in an interference signal such as surface EMG, it is virtually impossible, even with sophisticated algorithms, to distinguish the peripheral components of fatigue from the central ones. The conclusions of many authors on this topic should, therefore, be evaluated with caution and considered to be eminently speculative.

In conclusion, although some complexity indices seem to detect MMF efficiently, more work remains to be done to compare these indices in terms of reliability and sensibility, to optimize the choice of the parameters used to reconstruct the phase space and to elucidate their relationship with the physiologic phenomena underlying the onset of fatigue in exercising muscles.

## Figures and Tables

**Table 1:** Estimation parameters and fractal dimension (FD) in studies comparing fresh vs. fatigued muscles.

Authors, Year	Muscle	Boxes Number (Range)	Unit Box	FD
Meduri et al., 2016 [85]	BB	NA	−8–1.59	1.5 vs. 1.62
Mesin et al., 2009 [81]	VL	NA	1/640–1/40 of EMG time/amplitude size	0.4 vs. 0.6
Poosapadi et al., 2012 [82]	VL BB FDS	NA	NA	1.96 vs. 2.00
Gitter et al., 1995 [71]	BB	8–125	5580 μV/μs	1.1 vs. 1.4
Xu et al., 1997 [79] *	-	1–32	NA	1.1 vs. 1.8

* Contraction type was simulated in this work and isometric in all the others. BB = biceps brachii; FDS = flexor digitalis superficialis; VL = vastus lateralis; T = trapezius.

**Table 2:** Percent determinism (%DET) and percent recurrence (%REC) in fresh vs. fatigued muscles by RQA.

Authors.	Muscle	*m*	*τ* (ms)	r	%DET	%REC
Del Santo et al., 2007 [118]	D BB Q	15	3	10%	62 vs. 72 75 vs. 87 19 vs. 32	NA
Farina et al., 2002 [116]	BB	15	3–6	10% (^a^)	28 vs. 70	3.1 vs. 3.5
Felici et al., 2001a [126]	VL	15	τ_0_	2%	27 vs. 42	NA
Felici et al., 2001b [122]	BB	15	τ_0_	2%	33 vs. 78	NA
Fattorini et al., 2005 [115]	FD	15	τ_0_	2%	40 vs. 65	NA
Filligoi et al., 1999 [113]	BB	15	τ_0_	2%	36 vs. 60	4
Ikegawa et al., 2000 [123]	MF	10	τ_0_	2%	11 vs. 25	3.6 vs. 4
Ito et al., 2012 [127]	BB	-	-	10%	+15%	NA
Mesin et al., 2009 [78]	VL	7	1	20%	NA	
Schmied et al., 2011 [119]	EC	10	3	20%	43 vs. 50	
Uzun et al., 2012 [125]	BB, BR	6	4	-	20 vs. 60	
Webber et al., 1994 [111]	BB	10	τ_0_	2%	20 vs. 30	
Webber et al., 1995 [114]	BB	10	τ_0_	2%	20 vs. 40	
Webber et al., 2007 [112]	BB	10	4	15%	61 vs. na	
Yanli et al, 2005 [101]	BM	7	3	-	82 vs. na	
Yang et al., 2005 [124]	BB	10	4	15%	55 vs. 90	

*m* = embedding dimension; *τ* = delay; *τ*_0_ = first zero of the autocorrelation function (typically between 3–5 ms); *r* = radius as % of maximum distance or (^a^) of mean distance; BB = biceps brachii; BM = back muscles; BR = brachioradialis; D = deltoid; EC = extensor carpi radialis; FD = first dorsal interosseous; MF = multifidus; Q = quadriceps; VL = vastus lateralis.

**Table 3:** Entropy of sEMG during contractions.

Authors	Contraction	Muscle	Estimator	*r*	Value
Ahmad et al., 2008 [117]	Isometric	FC, EC	ApEn	4	0.5–0.79
Cashaback et al., 2013 [138]	Isometric	BB	MSE	0.60	0.9–1.2
Hernandez et al., 2019 [93]	Isometric Dynamic	VL	SampEn	0.20	1.46–1.57
Pethick et al., 2019 [91]	Isometric	VL	ApEn SampEn	0.10	0.10–0.65 0.01–0.62
Xie et al., 2010 [129]	Isometric	BB	ApEn FuzzyEn	0.10/0.15	0.0–3.0 0.4–0.8
Zhu et al., 2017 [134]	Isometric	BB	SampEn FuzzyEn	0.25	0.8–1.00 0.01–0.13

*r* = threshold expressed as a fraction of standard deviation; BB = biceps brachii; Q = quadriceps; VL = vastus lateralis; EC = extensor carpi radialis; FC = flexor carpi ulnaris.
